# Identification of the CFTR c.1666A>G Mutation in Hereditary Inclusion Body Myopathy Using Next-Generation Sequencing Analysis

**DOI:** 10.3389/fnins.2018.00329

**Published:** 2018-05-22

**Authors:** Yan Lu, Yu-Wei Da, Yong-Biao Zhang, Xin-Gang Li, Min Wang, Li Di, Mi Pang, Lin Lei

**Affiliations:** ^1^Department of Neurology, Xuanwu Hospital, Capital Medical University Beijing, China; ^2^Beijing Advanced Innovation Center for Big Data-Based Precision Medicine, Beihang University Beijing, China; ^3^Beijing Institute of Genomics, Chinese Academy of Sciences Beijing, China; ^4^School of Medical and Health Sciences, Edith Cowan University Joondalup, WA, Australia; ^5^Department of Neurology, Zhengzhou University People's Hospital Zhengzhou, China

**Keywords:** hereditary inclusion body myopathy, next-generation sequencing, CFTR, mutation, whole-exome sequencing

## Abstract

Hereditary Inclusion Body Myopathy (HIBM) is a rare autosomal dominant or recessive adult onset muscle disease which affects one to three individuals per million worldwide. This disease is autosomal dominant or recessive and occurs in adulthood. Our previous study reported a new subtype of HIBM linked to the susceptibility locus at 7q22.1-31.1. The present study is aimed to identify the candidate gene responsible for the phenotype in HIBM pedigree. After multipoint linkage analysis, we performed targeted capture sequencing on 16 members and whole-exome sequencing (WES) on 5 members. Bioinformatics filtering was performed to prioritize the candidate pathogenic gene variants, which were further genotyped by Sanger sequencing. Our results showed that the highest peak of LOD score (4.70) was on chromosome 7q22.1-31.1.We identified 2 and 22 candidates using targeted capture sequencing and WES respectively, only one of which as CFTRc.1666A>G mutation was well cosegregated with the HIBM phenotype. Using transcriptome analysis, we did not detect the differences of CFTR's mRNA expression in the proband compared with healthy members. Due to low incidence of HIBM and there is no other pedigree to assess, mutation was detected in three patients with duchenne muscular dystrophyn (DMD) and five patients with limb-girdle muscular dystrophy (LGMD). And we found that the frequency of mutation detected in DMD and LGMD patients was higher than that of being expected in normal population. We suggested that the CFTRc.1666A>G may be a candidate marker which has strong genetic linkage with the causative gene in the HIBM family.

## Introduction

Hereditary inclusion body myopathy (HIBM) refers to a rare heterogeneous group of neuromuscular diseases with autosomal recessive or dominant inheritance, which is characterized by muscle fibers containing rimmed vacuoles and inclusions consisting of tubulofilaments with a diameter of 15–21 nm (Askanas and Engel, 1993, 1998; Broccolini and Mirabella, 2015). The most common form of HIBM, also known as GNE myopathy, is autosomal recessive (Nonaka et al., 1981; Nishino et al., 2002), and the other identified forms of HIBM are autosomal dominant. This disorder of GNE myopathy is mainly due to the UDP-Nacetylglucosamine 2-epimerase/N-acetylmannosamine kinase (GNE) gene mutations, leading to abnormal sialylation of glycoproteins and relentless muscle degeneration (Argov and Yarom, 1984; Eisenberg et al., 2001). A rare subtype is HIBM with Paget's disease of the bone and frontotemporal dementia (HIBM-PFD), which is due to the valosin-containing protein (VCP) gene mutations, resulting in abnormal accumulation of ubiquitinated proteins and impaired autophagy in IBM-PFD muscle (Watts et al., 2004; Ju et al., 2009; Nalbandian et al., 2011). Moreover, autosomal dominant (AD) HIBM with congenital joint contractures and external ophthalmoplegia is associated with a mutation of the Myosin Heavy Chain IIa gene (MyHC-IIa), which causes a pathogenic effect through interfering with filament assembly or functional defects in ATPase activity (Darin et al., 1998; Martinsson et al., 2000). Despite advances in diagnosis and treatment, the heterogeneous group of HIBM still remains clinically underrecognized. Though a unique progression of muscle weakness with HIBM, most patients go un-diagnosed due to lack of clinical knowledge about this rare condition. In any case, early diagnosis would understand disease progress and help avoid unnecessary therapeutic options for these patients (Das et al., 2016).

Therefore, much needs to be learned about the other forms of HIBMs. Our previous study described a novel autosomal dominant HIBM in a Chinese HIBM family of Han descent. The clinical features of our family members are not compatible with any known autosomal dominant or recessive HIBM. With the exception of the mode of inheritance, the clinical phenotype of this new form of IBM is similar to an atypical adult onset HIBM2/DMRV which is characterized by muscle weakness and atrophy beginning in the distal muscles of the lower limbs with relative sparing of the quadriceps (Lu et al., 2012). Further sequencing excluded the mutations in GNE gene. Linkage analysis and haplotyping determined a new HIBM locus on chromosome 7q22.1-31.1 (Lu et al., 2012). To identify the corresponding causative gene defect in this muscle disorders, we herein performed targeted capture sequencing and whole-exome sequencing, and identified the c.1666A>G mutant in CFTR. The CFTR c.1666A>G mutant was well co-segregate with the HIBM phenotype in the verified members of the pedigree. After further Sanger sequencing on 101 patients with muscle diseases, the heterozygous c.1666A>G mutation in CFTR was identified in 8 cases. Our results provided characteristic etiology for the HIBM pedigree, and shed light on the corresponding pathogenic mechanisms.

## Materials and methods

### Linkage analysis

Our study was approved by the Institutional Ethics Committee of Xuan Wu Hospital, the Capital Medical University, and the written informed consents were obtained from all of the patients involved. We firstly removed erroneous and uninformative markers, then filtered markers if the distance of adjacent ones smaller than 0.1 centimorgans (cM) (Lu et al., 2012). In the present study, we performed multipoint linkage analysis using SUPERLINK (V1.1) with parameters as follows: Mode of inheritance, Dominant; multipoint window size, 3; Disease mutant gene frequency, 0.1, 0.01, or 0.0001; penetrance, 0.8, 0.8 or 0.99, 0.99.

### Targeted capture and whole-exome sequencing

**Figure 1:**
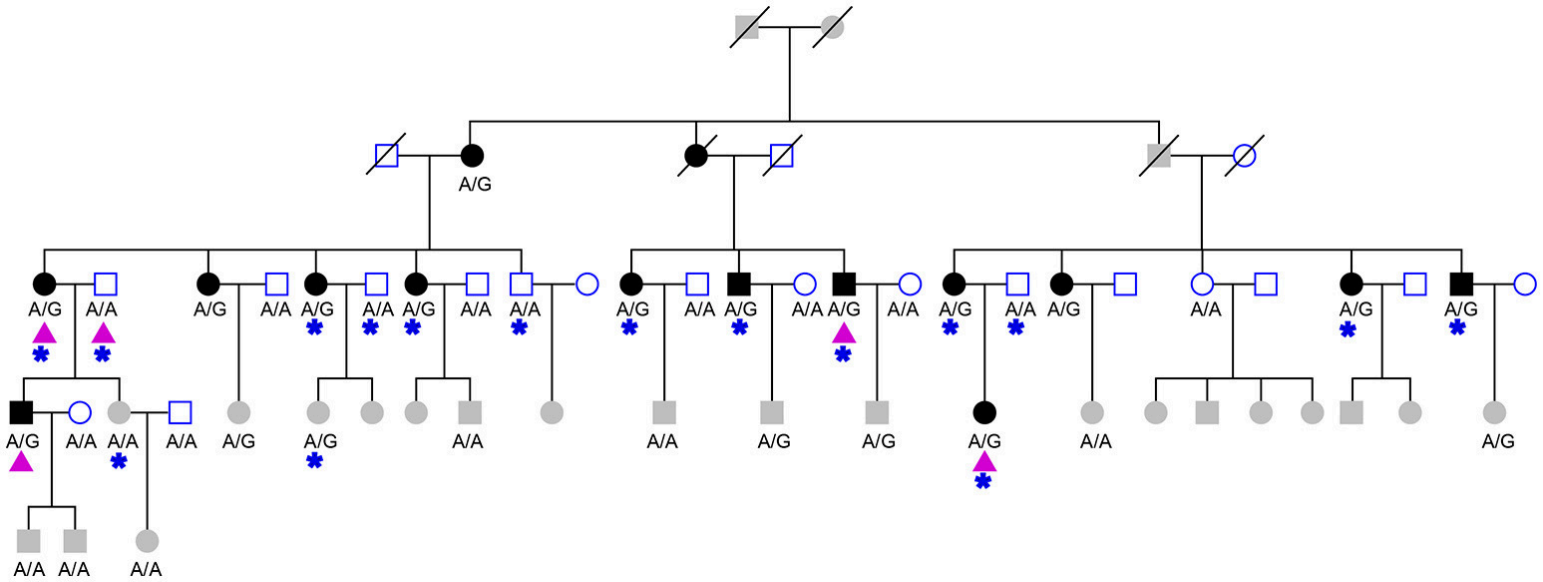
The studied HIBM pedigree. The pedigree consists of 27 males (squares) and 32 females (circles), 15 affected (solid) and 20 unaffected (hollow) members. Three deceased members (oblique line) of unknown phenotype and 21 members not old enough to determine phenotype (grayed squares and circles). The genotype of CFTRc.1666A>G is labeled under collected samples. Purple triangles indicate members of pedigree used for whole exome sequencing and blue stars indicate individuals used for targeted-capture sequencing.

A SureSelect targeted capture kit for a genomic region (chr7: 100310001-130180000, hg19), which contained the susceptibility locus was ordered from Agilent. DNA samples of 16 members of HIBM pedigree were captured according to the manufacturer's specifications (Figure 1). Because the disease does not appear until adulthood we did not include individuals from the pedigree who were under 30 when we chose members of the HIBM pedigree. Paired-end sequencing with 100-bp read length was conducted on each sample using IlluminaHiSeq2000 system (Illumina, San Diego, CA, USA). All paired reads were mapped to the human reference genome (hg19) using BWA (version 0.7.15) (Li and Durbin, 2010). PCR duplicates of the reads were removed using the Picard software program (version 1.92). The GATK (McKenna et al., 2010) (version 3.6) and Pindel (Ye et al., 2009) software packages were used to detect single nucleotide variants and genomic structural variants, respectively. The cn. MOPS package was used for copy number variation identifying (Klambauer et al., 2012).

For whole-exome sequencing, the Agilent SureSelect Human All Exon 50 Mb kit was used to capture whole exomes of 5 members of the HIBM pedigree (Figure 1). The next-generation sequencing assay and data analyzing were same as a forementioned targeted capture sequencing.

### Sanger sequencing for candidate variants of HIBM pedigree

We designed the primer sets for the 2 mutations from targeted capture sequencing and the 22 mutations from whole-exome sequencing using Primer-Premier 5.0 (PREMIER Biosoft International, Palo Alto, CA, USA). PCR reactions, products purifying, and Sanger sequencing were performed by ORI-GENE company. Sequence reads were analyzed using Phred/Phrap/Polyphred/Consed (University of Washington, Seattle, WA, USA) software and the genotype of variants were manually confirmed.

### Validating CFTRc.1666A>G in sporadic patients with muscular dystrophy

Considering the lower incidence of HIBM, we have no way to collect other pedigree of this new subtype of HIBM. So we collected additional 101 patients with muscle diseases to further verify the important roles of candidate variant in muscle pathology. The peripheral blood was drawn from all the individuals and genomic DNA was extracted. Using the primers aforementioned, sequencing reaction was also performed by ORI-GENE company and the frequency was further calculated.

### RNA extraction and illumina RNA-sequencing

Total RNA was isolated from the skeletal muscle tissue from patients III-1 and two healthy individuals from traffic injury using the Trizol reagent (Invitrogen, CA, USA). The eligible cDNA library was sequenced using the lluminaHiseq2000 platform by COMPASS biotechnology company. Differentially expressed genes (DEGs) were identified with FDR < 0.05. The protein-protein interaction (PPI) network between CFTR and DEGs was constructed using the information stored in BIOGRID databases and visualized by Cytoscape software (Shannon et al., 2003).

## Results

### Susceptibility locus identified from linkage analysis

**Figure 2:**
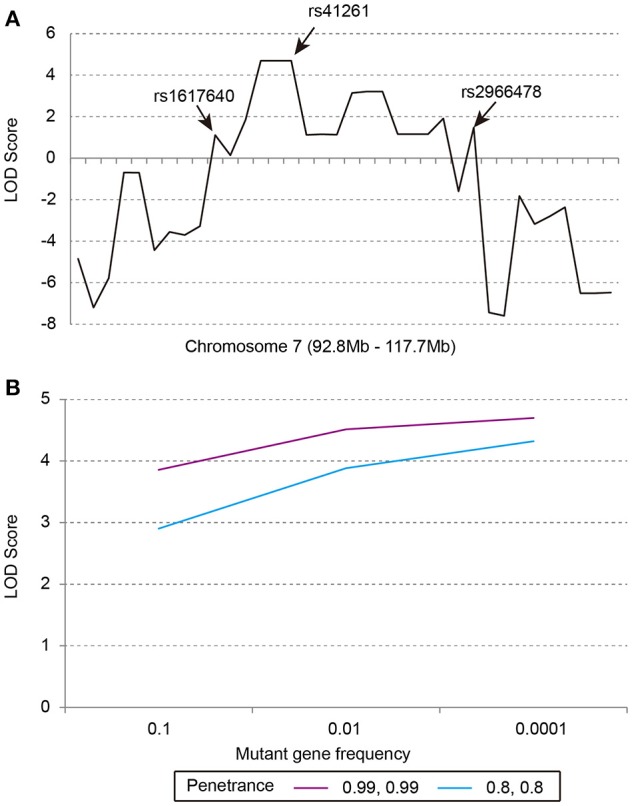
The multipoint parametric linkage scores of the studied pedigree. **(A)** The LOD scores of markers from 92.8 to 117.7 Mb on chromosome 7, rs1617640:EPO, rs41261:CDHR3, rs2966478:LOC101928012. **(B)** The LOD scores of rs41261 under the dominant inheritance mode with penetrance of 0.8, 0.8 or 0.99, 0.99 and mutant gene frequency of 0.1, 0.01, or 0.0001.

Our previous linkage analysis has identified a locus on chromosome 7q22.1-31.1 with a maximum multi-point LOD score of 3.65 for the studied pedigree (Figure 1) using MERLIN (Lu et al., 2012). However, limited by the computational memory needed by MERLIN, some offspring were removed from analyzing. Here, we employed a newly developed software SUPERLINK-Online who can handle large pedigree by drawing power from thousands of CPUs. Under the dominant inheritance mode, a multipoint analysis implement on the pedigree with several disease allele frequency and penetrance. We again found that the highest peak of LOD score was on chromosome 7q22.1-31.1, which was defined as a core region that ranged from rs1617640 (chr7:100317048, hg19) to rs2966478 (chr7:112260976, hg19) (Figure 2A). The top SNPrs41261 has the maximum LOD of 4.70 and 4.32 under penetrance of 0.99 and 0.8, respectively (Figure 2B). Frequency in the pedigree = Number of patients/All tested samples^*^2, Frequency in the pedigree = 23/37^*^2 = 0.31081.

### Identifying causal mutation using next-generation sequencing

**Figure 3:**
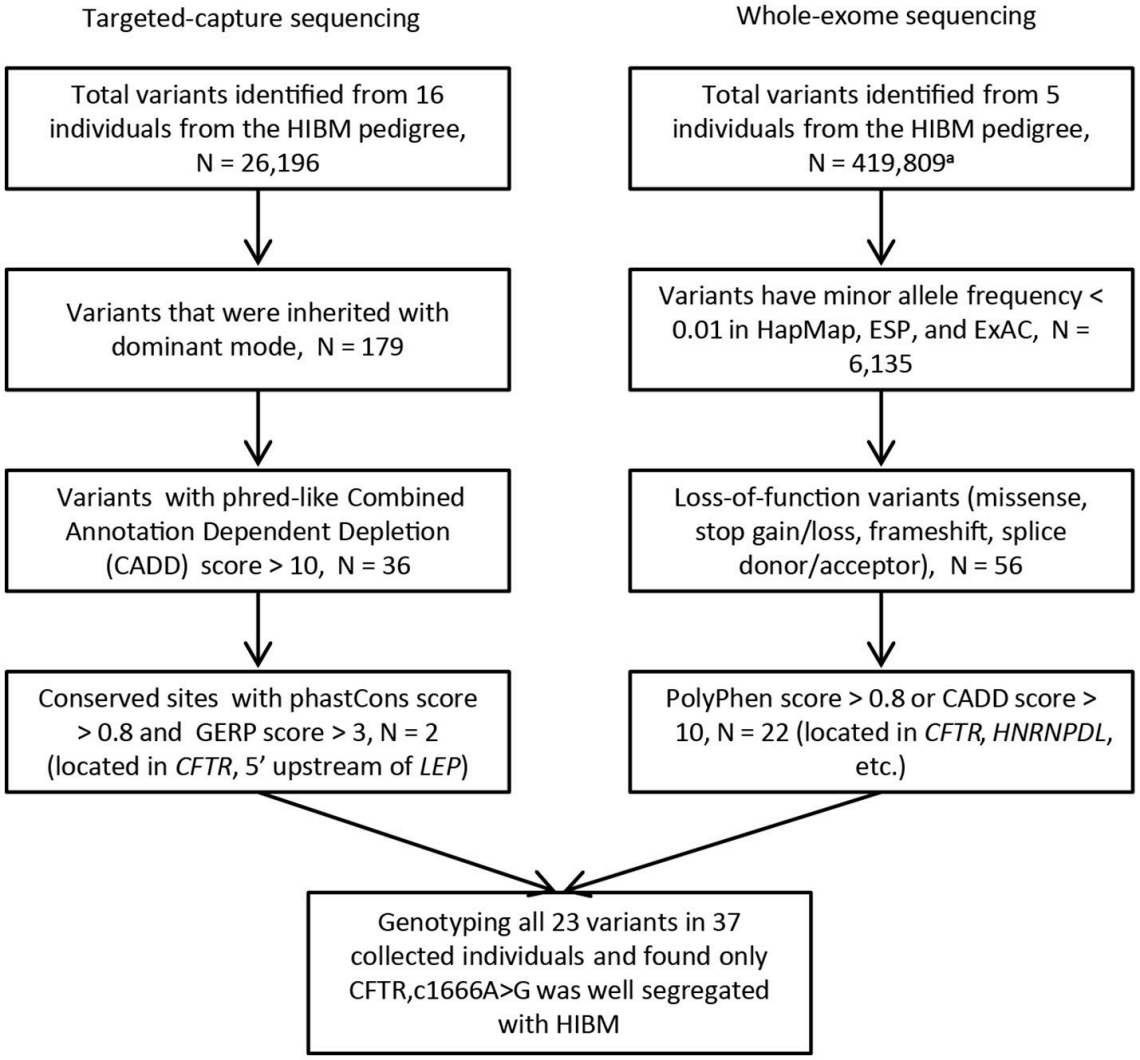
The flow chart of variants filtering. N, the number of variants; HIBM, hereditary inclusion body myopathy; phastCons score, base-by-base conservation score; GERP score, Genomic Evolutionary Rate Profiling score for producing position-specific estimates of evolutionary constraint; HapMap, the international haplotype map project; ESP, the NHLBI GO Exome Sequencing Project; ExAC, The Exome Aggregation Consortium. a. Variants are called without limited by the targeted regions.

In order to pinpoint causal mutation, we carried out the targeted capture sequencing on 16 samples from the HIBM pedigree (Figure 3). Considering causal mutation maybe located in the vicinity of the core region, we expanded the targeted region to chr7:100310001-130180000 as a predesigned region. In order to optimize the targeted region, we skipped the gene desert genomic regions and large intergenic regions when designing the probes for sequences outside the core region. In the end, the probes cover about 5.86 Mb genomic regions for the predesigned region.

Next-generation sequencing yielded an average coverage of 273.5X (standard deviation = 28.7) for the 5.86 Mb genomic region and identified 25,967 single-nucleotide variants (SNVs), 222 genomic structure variants (SVs), and 7 copy number variation regions (CNVRs). After a process of variants filtering, we finally obtained 2 candidates for the pedigree: c.1666A>G (p.I556V) located in the exon 12 of *CFTR*, rs6960959 located in a conserved region at the upstream of *LEP*(Leptin). We genotyped the rest of collected family members and found only c.1666A>G well segregated with HIBM in the pedigree. We examined the c.1666A>G in SNP databases and found that the global minor allele frequency is 0.0112 in 1000 Genomes project (1KG) and CFTRc.1666A>Gwas not detected in the NHLBI GO Exome Sequencing Project (about 6500 samples). The candidate mutation of CFTRc.1666A>G was further evaluated by investigating its prevalence in the public database curated by the Genome Aggregation Database (gnomAD), which spans 123,136 exome sequences. The global minor allele frequency of CFTRc.1666A>G is 0.003306 in the database of gnomAD. Moreover, the allele frequency of CFTRc.1666A>G is 0.00291 in ExAC.

### Validating CFTRc.1666A>G using whole-exome sequencing

In order to verify the CFTR c.1666A>G as a biomarker which has strong linkage disequilibrium with the causative mutation in the HIBM family, we performed whole-exome sequencing on 5 individuals from the HIBM pedigree (Figure 1). A total of 419,809 variants were identified from the whole-exome of 5 individuals and 22 variants were picked out as candidates after a series of variants filtering, which contained the c.1666A>G mutant in CFTR (Figure 3). After genotyping these 22 variants in the HIBM pedigree, HNRPDLc.1332C>T was segregated well with the 5 whole-exome sequencing individuals, while it was not well segregated with the phenotype in our pedigree. Moreover, we found that CFTRc.1666A>G mutant was the only one that was well segregated with the phenotype.

The CFTR protein consists of five domains. There are two transmembrane domains, each with six spans of alpha helices. These are each connected to a nucleotide binding domain (NBD) in the cytoplasm. The first NBD is connected to the second transmembrane domain by a regulatory “R” domain that is a unique feature of CFTR. The ion channel opens only when its R-domain has been phosphorylated by PKA and ATP is bound at the NBDs. We further performed the protein structure prediction using the Protein Structure Prediction Server (PSIPRED). The p.I556V is located in α-helical-rich conformation. The wild type 556I forms hydrogen bonds with the adjacent AA in the alpha-helix domain. Similarly, the mutant 556 V form hydrogen bonds with the adjacent AA in the alpha-helix domain. However, the free energy is much lower in mutant compared with wild type, and the conformation seems more stable in mutant compared with wild type.

### CFTRc.1666A>G in sporadic patients with muscular dystrophy

The results of linkage analysis, targeted-capture sequencing, whole-exome sequencing and genotyping indicated that the CFTRc.1666A>G is the most probable contributor to the HIBM phenotype of this pedigree. We suggested that CFTRc.1666A>G may be closely associated with muscle pathology and further sanger sequencing on 101 patients with various muscle diseases indicated that 8 cases are heterozygous for CFTRc.1666A>G mutation and the mutation frequency was about 0.04, suggesting the important roles of CFTRc.1666A>G in muscle pathology. Of the 8 patients, three patients were with duchenne muscular dystrophyn (DMD), which are characterized by progressive muscle weakness, cardiomyopathy, and respiratory failure in addition to cognitive impairment. The other five patients were with limb-girdle muscular dystrophy (LGMD), typically characterized by progressive weakness and wasting of the shoulder and pelvic girdle muscles. Totally, the 8 patients shared similarities of muscle weakness with the affected individuals of HIBM family in clinical phenotype.

### The global transcriptome profiling of HIBM

**Figure 4:**
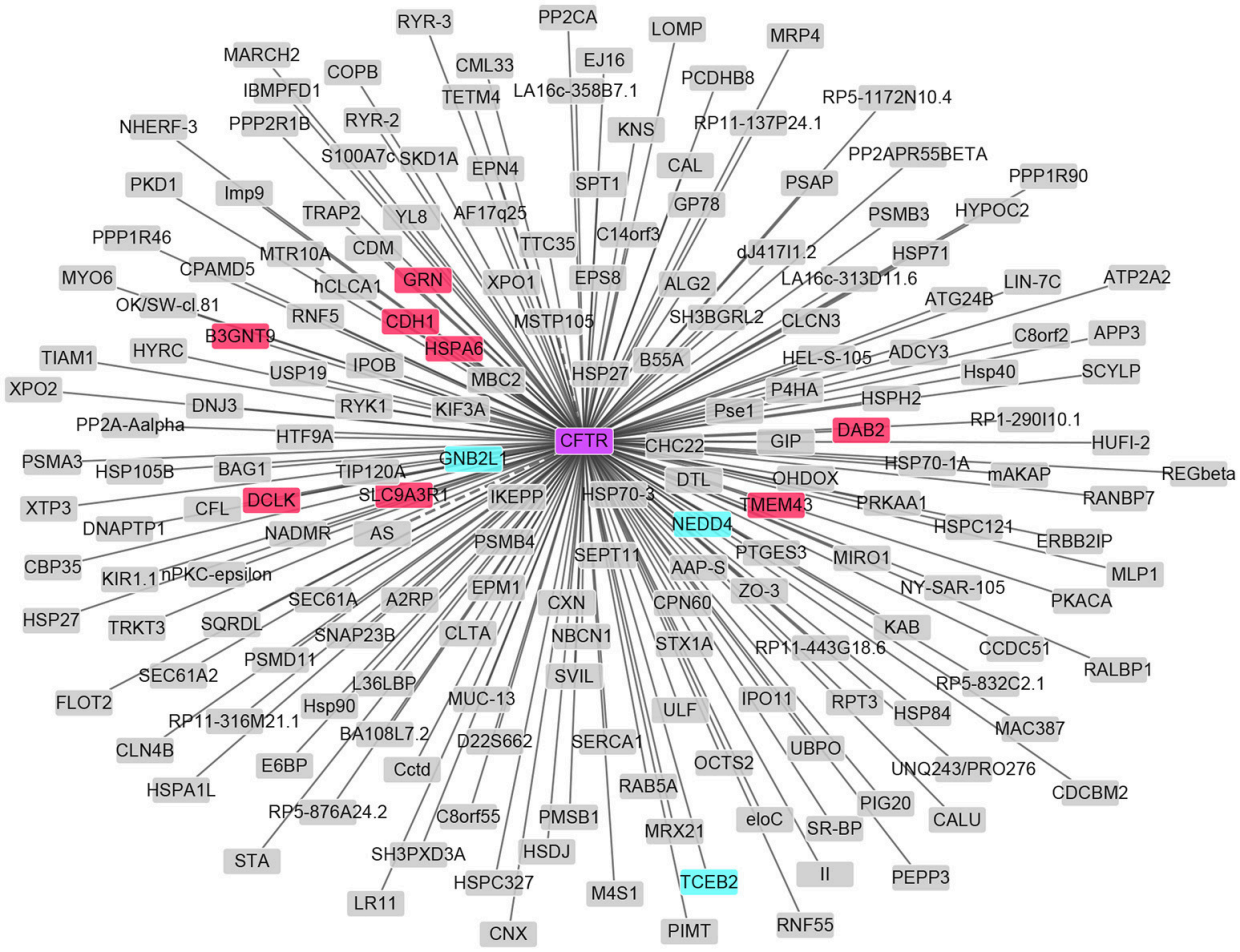
PPI network of CFTR and DEGs in HIBM. The up-regulated DEGs are in red and those down-regulated are in green.

As in Table S1, 1651 differentially expressed genes (DEGs) in total were identified in patients with HIBM compared with the healthy individuals, including 1086 up-regulated DEGs and 565 down-regulated DEGs. MYH8 and FAM166B were the most significant up-regulated and down-regulated DEGs, respectively. MYH8 encodes myosin heavy chain 8 with ATPase activity and act in filament binding, which functions in skeletal muscle contraction. However, we found that the mRNA expression of CFTR is not changed. The established PPI network showed that 11 differentially expressed genes interacted with CFTR. Among these genes, GRN, CDH1, HSPA6, SLC9A3R1, B3GNT9, DCLK, DAB2, and TMEM43 were up-regulated. GNB2L, NEDD4, and TCEB2 were down-regulated as showed in Figure 4.

## Discussion

The HIBMs are composed of a group of rare muscle disorders with different etiologies and clinical presentations (Askanas and Engel, 1993). There are mainly five different HIBMs, such as HIBM1, HIBM2, HIBM-PFD, HIBM-ERF, and HIBM3. Numerous studies have been performed and a few causal mutations had been reported (Nonaka et al., 1981; Horowitz and Schmalbruch, 1994; Darin et al., 1998; Goldfarb et al., 1998; Martinsson et al., 1999, 2000; Saavedra-Matiz et al., 2000; Nishino et al., 2002). Besides, our previous study described a new subtype of HIBM named as HIBM4. A novel susceptibility region was identified, which was located on chromosome 7q22.1-31.1 (Lu et al., 2012). Considering that gene mutations are the pathological basis of HIBM, we identified the candidate CFTR gene responsible for the phenotype in HIBM family by using targeted capture sequencing and whole-exome sequencing to elucidate the specific pathogenic mechanism and therapeutic perspectives of this novel subtype of HIBM.

Further linkage studies by SUPERLINK-Online software showed the same results as previous, suggesting that the susceptibility locus was located at 7q22.1-31.1. According to the prediction results of CFTR protein, we speculated that p.I556V in the CFTR may affect the channel from functioning properly, leading to a blockage of the movement of salt and water into or out of cells. To further verify whether the CFTR is causative gene, we performed targeted capture sequencing on 16 subjects and whole-exome sequencing on 5 individuals, indicating that CFTRc.1666A>G (p.I556V) was well segregated with HIBM in the verified pedigree. Moreover, the CFTRc.1666A>G mutation was also detected in 8 of the 101 patients with muscle diseases. But we did not detect the change in the expression level of CFTR after RNA-seq. There is no sufficient evidence to conclude that CFTR is causative gene, we hypothesized that CFTR mutation is a marker with strong linkage disequilibrium with the causative gene for HIBM4. We also established PPI network based on CFTR and found 11 DEGs among them. The 11 DEGs have no relationship with HIBM after retrieve.

In order to explore the causative gene that is linked to the CFTR, we retrieved the function of 40 genes in the upstream and downstream of the CFTR gene within 10 Mb. Interestingly, two of them as Interferon related developmental regulator 1(IFRD1) and Protein phosphatase 1 regulatory subunit 3A (PPP1R3A) were deserved to be mentioned. IFRD1 is an immediate early gene that encodes a protein related to interferon-gamma (Buanne et al., 1998). Mutations in this gene are related to sensory/motor neuropathy with ataxia. This gene may also be involved in modulating the pathogenesis of cystic fibrosis^1^. A previous study reported a single missense variant c.514 A>G in IFRD1 gene that was associated with Spinocerebellar ataxias (SCA18), which is an autosomal dominant sensory/motor neuropathy with ataxia. Symptoms included features of motor and sensory neuropathy, ataxia, pyramidal tract signs, dysmetria, and muscle weakness (Lin et al., 2018). However, the RNA-seq results showed no significant change of IFRD1 expression in skeletal muscle tissue of patients with HIBM4 compared with normal control.

PPP1R3A and the glycogen-associated form of protein phosphatase-1 (PP1) derived from skeletal muscle is a heterodimer composed of a 37-kD catalytic subunit and a 124-kD targeting and regulatory subunit. This gene encodes the regulatory subunit which binds to muscle glycogen with high affinity, thereby enhancing dephosphorylation of glycogen-bound substrates for PP1 such as glycogen synthase and glycogen phosphorylase kinase (Montori-Grau et al., 2007). Stored glycogen is an important source of energy for skeletal muscle. It is well known that human genetic disorders primarily affecting skeletal muscle glycogen turnover. Savage et al. (2008) identified a PPP1R3A FS (frameshift (FS) premature stop mutation in PPP1R3A (C1984DAG; stop codon 668; referred to subsequently asPPP1R3A FS variant), which encodes a truncated protein that is mistargeted within the cell. This mutation decreases muscle glycogen synthase activity, thereby decreasing muscle glycogen content in humans and mice (Savage et al., 2008). In the present study, we found that PPP1R3A was located at chromosome7q22.1-31.1. Moreover, PPP1R3A was significantly differentially expressed in skeletal muscle tissue of patients with HIBM4 compared with normal control. So we speculated that PPP1R3A may be closely related to the pathogenesis of HIBM4.

Taken together, our exome trapping studies suggested that the CFTRc.1666A>G may be a candidate marker which has strong linkage disequilibrium with the causative gene in the HIBM4 family. By scanning the upstream and downstream regions of the CFTR gene, we found that PPP1R3A was closely related to the HIBM. Therefore, we speculated that PPP1R3A may be the potential candidate causative gene for the HIBM4 pedigree. Our results will be helpful to better understand the molecular mechanisms and the pathogenesis of HIBM4.

Even so, our study has some limitations. Unfortunately we were not able to identify mutations in PPP1R3A by re-analyzing the data of targeted sequencing and WES, which is mainly due to the deficiency of capture technology in deep (next-generation) sequencing technologies, including the coverage of different exome databases, target coverage efficiency, GC bias, sensitivity in single nucleotide variant detection, etc. In future study, the genomic DNA will be extracted and the entire coding region and the intron-exon boundaries of PPP1R3A gene will be sequenced to confirm whether PPP1R3A might be the gene responsible for HIBM4 pedigree.

## Author contributions

YL and Y-WD: Conception and design; Y-BZ: Administrative support; X-GL, MW, and LD: Materials and samples available; MP: Data collection and collation; LL and Y-WD: Data and interpretation.

### Conflict of interest statement

The authors declare that the research was conducted in the absence of any commercial or financial relationships that could be construed as a potential conflict of interest. The reviewer CH and handling Editor declared their shared affiliation.

## Footnotes

^1^IFRD1 interferon-related developmental regulator 1 [Homo sapiens (human)][J].
