# Corrigendum: Identification of the CFTR c.1666A>G Mutation in Hereditary Inclusion Body Myopathy Using Next-Generation Sequencing Analysis

**DOI:** 10.3389/fnins.2018.00570

**Published:** 2018-08-22

**Authors:** Yan Lu, Yu-Wei Da, Yong-Biao Zhang, Xin-Gang Li, Min Wang, Li Di, Mi Pang, Lin Lei

**Affiliations:** ^1^Department of Neurology, Xuanwu Hospital, Capital Medical University, Beijing, China; ^2^Beijing Advanced Innovation Center for Big Data-Based Precision Medicine, Beihang University, Beijing, China; ^3^Beijing Institute of Genomics, Chinese Academy of Sciences, Beijing, China; ^4^School of Medical and Health Sciences, Edith Cowan University, Joondalup, WA, Australia; ^5^Department of Neurology, Zhengzhou University People's Hospital, Zhengzhou, China

**Keywords:** hereditary inclusion body myopathy, next-generation sequencing, CFTR, mutation, whole-exome sequencing

An error was found in the first and second sentence of the original article's abstract.

It had originally been published as:

Hereditary inclusion body myopathy (HIBM) is a rare autosomal recessive adult onset muscle disease which affects one to three individuals per million worldwide. This disease is autosomal dominant and occurs in adulthood.

The corrected sentences should read:

Hereditary Inclusion Body Myopathy (HIBM) is a rare autosomal dominant or recessive adult onset muscle disease which affects one to three individuals per million worldwide. This disease is autosomal dominant or recessive and occurs in adulthood.

The authors apologize for this error and state that this does not change the scientific conclusions of the article in any way.

The original article has been updated.

## Conflict of interest statement

The authors declare that the research was conducted in the absence of any commercial or financial relationships that could be construed as a potential conflict of interest.

